# Feasibility of using Determine TB-LAM to diagnose tuberculosis in HIV-positive patients in programmatic conditions: a multisite study

**DOI:** 10.1080/16549716.2019.1672366

**Published:** 2019-10-15

**Authors:** Sekai Chenai Mathabire Rucker, Loide Cossa, Rebecca E. Harrison, James Mpunga, Sheila Lobo, Patrick Kisaka Kimupelenge, Felix Mandar Kol’Ampwe, Isabel Amoros Quiles, Lucas Molfino, Elisabeth Szumilin, Oleksandr Telnov, Zibusiso Ndlovu, Helena Huerga

**Affiliations:** a Epicentre, Paris, France; b Médecins Sans Frontières, Chiradzulu, Malawi; c Médecins Sans Frontières, Maputo, Mozambique; d Médecins Sans Frontières, Kinshasa DRC; e National TB Control Program Malawi, Lilongwe; f Ministry of Health, Maputo, Mozambique; g Médecins Sans Frontières, Lilongwe, Malawi; h Médecins Sans Frontières, Paris, France; i Médecins Sans Frontières, Geneva, Switzerland; j Medecins Sans Frontières, Southern Africa Medical Unit, Cape Town, South Africa

**Keywords:** Lipoarabinomannan, implementation, tuberculosis, diagnosis, low income settings

## Abstract

**Background**: Determine TB-LAM is a urine-based point-of-care assay for diagnosis of tuberculosis (TB).

**Objective**: To assess the feasibility of using LAM to diagnose TB in adult HIV-positive patients in resource-limited settings.

**Methods**: We performed a multi-centric mixed-methods cross-sectional descriptive study in the Democratic Republic of Congo, Malawi, and Mozambique. We used the study and program monitoring tools to estimate user workload, turn-around time (TAT), and proportion of patients with LAM and sputum-based results. We conducted semi-structured interviews to assess the user acceptability of the LAM.

**Results**: The duration of the LAM testing activity per patient was 27 min (IQR 26–29); staff continued with other duties whilst waiting for the result. More patients had a LAM versus a sputum-based result: 168/213 (78.9%) vs 77/213 (36.1%), p < 0.001 in DRC; 691/695 (99.4%) vs 429/695 (61.7%), p < 0.001 in Malawi; and 646/647 (99.8%) vs 262/647 (40.5%), p < 0.001 in Mozambique. The median TAT in minutes when LAM was performed in the consultation room was 75 (IQR 45–188) in DRC, 29 (IQR 27–39) in Malawi, and 36 (IQR 35–41) in Mozambique. In comparison, the overall median TAT for sputum-based tests (smear or GeneXpert) was 2 (IQR 1–3) days. The median time to the first anti-TB drug dose for LAM-positive patients was 155 (IQR 90–504) minutes in DRC and 90 (IQR 60–117) minutes in Mozambique. The overall inter-reader agreement for the interpretation of the LAM result as positive or negative was 98.9%, kappa 0.97 (95%CI 0.96–0.99). Overall, LAM users found the test easy to perform. Major concerns were use of the reading card and the prior requirement of CD4 results before LAM testing.

**Conclusion**: It is feasible to implement the LAM test in low resource settings. The short TAT permitted same day initiation of TB treatment for LAM-positive patients.

## Background

Tuberculosis (TB) is the leading cause of morbidity and mortality in HIV-positive people, accounting for 32% of reported deaths in 2017 [1]. In resource-limited settings, postmortem data from HIV-positive patients have shown that TB was responsible for 40% of the deaths, of which 46% were undiagnosed [2]. TB diagnosis is difficult in resource-limited countries due to the lack of diagnostic tools [3]. An additional challenge for HIV-positive individuals is that frequently available diagnostic tools such as sputum smear microscopy perform poorly in this population [4].

The Determine TB LAM Ag test (Determine TB-LAM Ag test, Abbott, Waltham, MA, USA [formerly Alere]; LAM test) is a lateral flow assay for urinary Lipoarabinomannan (LAM) that can detect active TB within 30 min. LAM is useful in HIV-positive individuals with advanced immunodeficiency [5,6]. The World Health Organization (WHO) currently recommends the use of the LAM test to assist in the diagnosis of TB in HIV-positive patients with CD4 counts less than or equal to 100 cells/µl or in severely ill HIV-positive patients, regardless of the CD4 count, or in those with an unknown CD4 count [7]. Nonetheless, the operational aspects of introducing LAM for TB diagnosis in programmatic conditions have not been evaluated. The aim of this study was to assess the feasibility of using the LAM test to diagnose TB in adult HIV-positive patients in resource-limited settings.

## Methods

### Study setting and intervention

The feasibility assessment was conducted between February 2016 and September 2017 in the Democratic Republic of Congo (DRC), Malawi and Mozambique. The LAM test was assessed in the Centre Hospitalier de Kabida (CHK) in Kinshasa, DRC; the Chiradzulu District Hospital and Namitambo, Milepa and Mauwa rural primary health centres (PHCs) in Chiradzulu District, Malawi; and in the Centre of Reference in Alto-Maé (CRAM) and Primeiro de Maio urban PHCs in Maputo, Mozambique. Médecins Sans Frontières (MSF) supported the Ministry of Health (MoH) HIV and TB programs at these sites. These sites were selected as they were contexts in which the LAM test was already in use before the feasibility and prospective diagnostic study (DRC and Mozambique), or was planned to be used before the prospective diagnostic study (Malawi). The LAM test was performed at the patient bedside and in the consultation room in DRC. In Malawi, the LAM was initially performed at the district hospital laboratory, then later at patient bedside and in the consultation room. In Mozambique, the LAM was performed both in the laboratory and in the consultation room. The LAM assay was performed in HIV-positive adult patients. In DRC, the LAM was used for routine patient care in the regular HIV program. In Malawi, the LAM was introduced as part of a longitudinal study that aimed to assess the incremental diagnostic yield of TB diagnostic strategies, including the LAM test [8,9]. In Mozambique, the test was used in the regular HIV program and later as part of the same longitudinal study conducted in Malawi. In DRC, the test was performed on inpatients and outpatients with either a CD4 ≤100 cells/µL, a Karnofsky scale of <60, or with signs or symptoms of TB. In Malawi, LAM was performed on inpatients regardless of TB symptoms and on outpatients presenting with signs or symptoms of TB. In Mozambique, the test was performed on patients in the CRAM with a CD4 <100 cells/µL regardless of TB symptoms, and on patients in Primeiro de Maio with signs or symptoms of TB and CD4 <200. CD4 testing was planned to be performed at the same time as the LAM test. The LAM was performed by doctors, nurses, clinical officers or laboratory technicians. Following the manufacturer’s specifications, the LAM was performed by applying 60 µL of freshly collected urine to the LAM test strip. The results of the test were recorded as positive, negative or invalid. For positive results, the grade of intensity was also recorded using the four bands graded intensity scale.

### Study design and data collection

We conducted a mixed methods multi-centric cross-sectional descriptive study using quantitative and qualitative methods.

For the quantitative part of the study, patient data was collected in Malawi and Mozambique as part of a longitudinal study and the sample size was calculated according to the main objective of the study [9]. In DRC, the sample size was considered as all the patients who met the criteria for a LAM test as described earlier, during the period from July to September 2017. Health workers in DRC performed a double reading of the LAM result and documented the time of each step in the LAM testing procedure. The number of eligible patients who had a LAM and/or sputum-based test results was extracted from the patient admissions register.

We used patients’ individual data from the longitudinal study conducted in Malawi and Mozambique and routinely collected data in DRC to evaluate the LAM test inter-reader agreement, the proportion of patients with a LAM result and sputum-based test results (microscopy or GeneXpert assay: Cepheid, Sunnyvale, CA, USA; GeneXpert), and the LAM result turnaround time (TAT). Longitudinal data were collected using case report forms, and subsequently recorded in Epi-Data 3.0 software (The Epi-Data Association, Odense Denmark) whilst the routinely collected data was recorded using Microsoft Excel.

The qualitative part of the study included semi-structured interviews and observation of health workers performing the LAM test and of the workspace environment. Purposive sampling was used to include all health workers who had directly performed the LAM test. The health workers were invited to participate in one-on-one interviews conducted by the lead author (SCMR) and also to fill in a written questionnaire anonymously, see in Appendix Annex 1 and Annex 2. Program managers directly involved in the LAM test implementation and health workers directly encountered in the TB diagnosis process were also purposively included in the study. In total, 49 staff participated in the assessment (17 in DRC, 22 in Malawi, 10 in Mozambique), Table 1. Among them, 32 had performed the LAM test. Closed and open-ended questions were used in the written questionnaire and one-on-one interviews to assess the LAM test acceptability and its perceived strengths and challenges as well as the aspects of the test related to its programmatic implementation. We asked broad questions such as: ‘What do you know about the LAM test?’ and more specific questions such as ‘What are your views on performing the LAM test relating to your daily workload?’, and ‘What are you views in relation to the introduction of the LAM test as a TB diagnostic tool?’ Further questions were asked to understand the context in which the LAM test was being implemented. Follow up questions were used during the interview to probe for more information.

**Table 1.** Health workers who participated in the LAM test feasibility assessment.

Position of health worker	DRC		Malawi		Mozambique		Total
	MSF	MOH	MSF	MOH	MSF	MOH	
Laboratory technician	0	0	6	0	2	0	8
Nurses	3	0	3	3	2	0	11
Clinical officers	0	0	4	1	2	0	7
Doctors	9	0	0	0	1	0	10
District medical officer	0	0	0	1	0	0	1
Environmental health technicians	0	0	0	1	0	1	2
Health assistants/microscopists	0	2	0	3	0	0	5
Program Managers	3	0	0	0	2	0	5
Total	15	2	13	9	9	1	49

To assess the workspace changes made for the implementation of the LAM, we observed, took pictures and asked questions regarding the previous and current setup of the rooms, and documented this in the study diary. To assess the changes in patient flow related to the LAM, we checked the existing records relating to the TB diagnostic algorithms and compared the steps in the diagnostic algorithms prior to and after the introduction of the LAM. From this assessment, we theoretically deduced the number of health center visits made by the patient during the TB diagnosis process, before and after LAM introduction. The actual number of visits made by patients was not measured.

The lead author also conducted field visits to all the study sites, observing the staff as they performed the LAM test and observing clinic processes relating to the TB patient flow, including diagnosis and treatment of TB.

### Data analysis

Responses to the closed-ended questions were compiled in an Excel sheet and summarised as counts and percentages. Interview notes, responses to open-ended questions in the written questionnaires, and the field notes were inductively and manually coded. After coding, thematic analysis was performed following the method outlined by Braun and Clarke [10]. We considered acceptability as the extent to which the LAM users considered the LAM test to be appropriate, based on their experiences and perceptions [11]. The daily proportion of the staff workload used on performing the LAM was calculated as the sum of the time in minutes expended on each patient in 1 day divided by 420 min (assuming a seven-hour working day) and multiplied by 100. The time to instruct the patient on how to collect the urine sample plus the time interval from the initiation of the test until the reading of the result was also considered.

The inter-reader agreement and its kappa value (95% Confidence Intervals) were calculated to determine consistency among observers. The TAT for the LAM test was considered as the minutes elapsed from the time the urine was requested by the clinician to the time the clinician received the results. This data were summarized by median and interquartile range. The count and proportion of patients who had a LAM result and a sputum-based result were compared using the chi-squared test. We used Stata 13 (Stata Corporation, College Station, Texas, USA) for the analyses. The duration of the TB diagnostic procedure was theoretically deduced as the number of visits made by the patient from the first day of consultation or hospitalization to the day a TB diagnosis was made or excluded.

### Ethical considerations

Ethics approval was obtained from Médecins Sans Frontières Ethical Review Board and from the National Ethical Review Committees in DRC, Malawi and in Mozambique. Eligible staff gave verbal consent to participate after being informed about the study; they were also informed of their right to decline participation at any time. Written consent was sought from patients who participated in the longitudinal LAM studies in Malawi and Mozambique. In DRC, no consent was sought from patients as we used retrospectively anonymized individual data routinely collected within the program.

## Results

### Changes in patient flow and duration of diagnostic procedures

**Figure 1.**
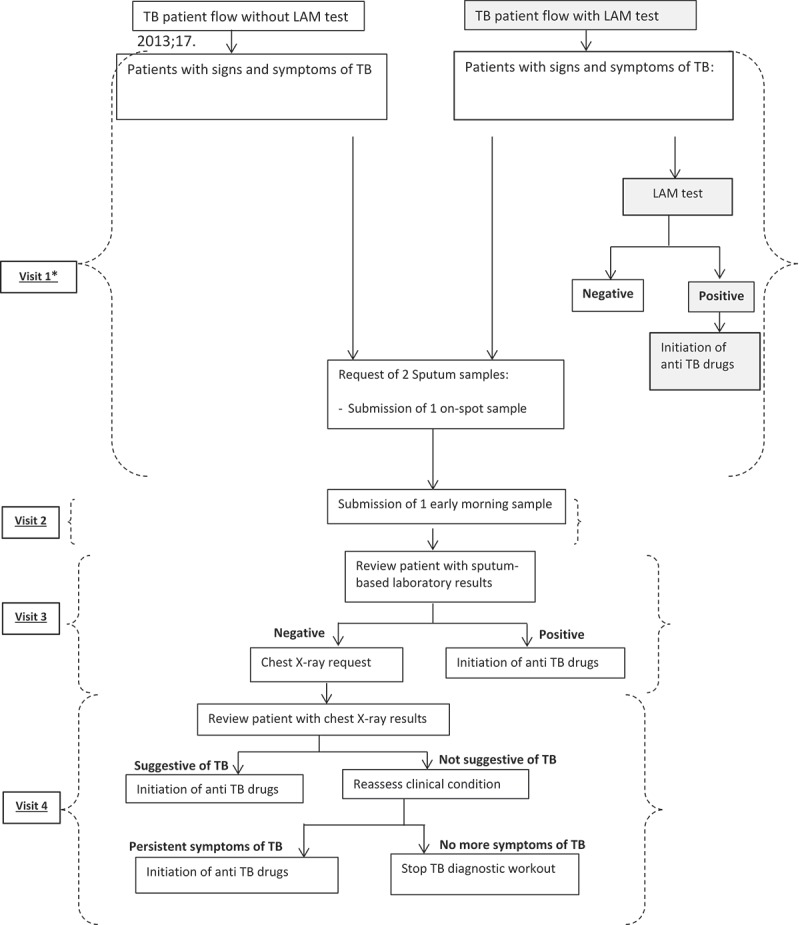
Comparison of TB diagnosis procedures in HIV-positive patients with signs and symptoms of TB before and after the introduction of the LAM test. *Patients could be initiated on TB treatment based on clinical criteria before and after the LAM test was introduced.

Following the TB diagnostic algorithm without the LAM, patients would, in theory, be required to make up to four visits to the health facility before a TB diagnosis is made (Figure 1). When the LAM is added to the TB diagnostic algorithm, the number of visits before starting TB treatment could potentially be reduced to one visit, if the LAM result were positive. However, sputum samples for microscopy and Xpert MTB/RIF would still be requested despite a LAM-positive result and being already on TB treatment, so patients would still be required to return the following day with the early morning sputum sample. Patients with a LAM-negative result would follow the regular routine procedures, so the number of visits probably would not change for these patients.

### Workload and logistical implications related to the use of LAM

The median time used to instruct the patients on urine collection was 2 (IQR 1–5) minutes, whilst the time to perform the LAM test was 25 (IQR 25–27) minutes. The median time used for the LAM activity per patient per staff was 27 (IQR 26–29) minutes. A median of 2 (IQR 1–3) LAM-eligible patients were seen daily, which meant median 54 (IQR 32–78) minutes were spent daily on the LAM. This accounted for 12.9% of the daily work time. However, this estimate does not consider that health staff continued to perform other duties while waiting to read the LAM test result.

Additional equipment required to perform the LAM test were 60 ml sterile containers for urine collection, single-use-graduated pipettes, and a timing device. No extra space was required to perform or to store the test, as it was performed in the consultation rooms or at the patient’s bedside and could be easily stored in the existing storage rooms.

### LAM result availability, turn-around-time and inter-reader agreement

Data from 1555 patients were used in the analysis (213 from DRC, 695 from Malawi and 647 from Mozambique). The number of patients who were able to produce urine versus those who could provide a sputum sample were 168/213 (78.9%) vs 168/213 (78.9%) p insignificant in DRC; 691/695 (99.4%) vs 535/695 (77.0%), p < 0.001 in Malawi; and 646/647 (99.8%) vs 262/647 (40.5%), p < 0.001 in Mozambique. The only patients for whom urine samples could not be collected were very sick inpatients who had died before a sample could be collected. Because of the relative ease of obtaining a sample, more patients were able to have a LAM test result than had sputum-based result (microscopy or GeneXpert): 168/213 (78.9%) vs 77/213 (36.1%), p < 0.001 in DRC; 691/695 (99.4%) vs 429/695 (61.7%), p < 0.001 in Malawi; and 646/647 (99.8%) vs 262/647 (40.5%), p < 0.001 in Mozambique.

The LAM had a shorter TAT compared to the other TB diagnostic tests. The delays in performing the LAM test were mainly due to high patient loads per staff and patients with difficulties producing urine. Overall, patients took a median of 7 (IQR 5–20) minutes to submit a urine sample. The median TAT when LAM was performed in the consultation room was 75 (IQR 45–188) minutes in DRC, 29 (IQR 27–39) minutes in Malawi, and 36 (IQR 35–41) minutes in Mozambique. The median TAT when LAM was performed in a laboratory was 196 (IQR 119–247) minutes in Malawi and 45 (IQR 40–51) minutes in Mozambique. In comparison, the median TAT for sputum-based tests (smear or GeneXpert) was 2 (IQR 1–3) days. Data for TAT from the laboratory was not available in DRC. Patients with LAM positive results were able to start TB treatment on the same day as the LAM result. The median time from LAM request to the time the first dose of anti-TB drugs was taken was 155 (IQR 90–504) minutes in DRC and 90 (IQR 60–117) minutes in Mozambique. This data was not available in Malawi.

The overall inter-reader agreement for the LAM test graded result was 94.2%, kappa 0.91 (95%CI: 0.89–0.92) and for LAM interpretation as positive or negative was 98.9%, kappa 0.97 (95%CI 0.96–0.99).

### Staff perceptions on the use of LAM

Overall, three themes emerged relating to the advantages of using the LAM test: first, the rapidity of the test; second, the ease with which the test could be performed; and third, the relevance of the LAM in the settings in which it was implemented.

Most LAM users perceived the LAM as giving fast TB diagnosis results, and consequently leading to rapid initiation of anti-TB treatment: ‘It’s quick and easy to do the test and make a decision, before the patient leaves’ [Nurse, DRC]. Performing the LAM test in the consultation room or at the patient bedside was considered as more advantageous compared to performing it in a more central location such as the district laboratory: ‘Patients avoid being sent to another place in the health centre and can wait for their results instead of being asked to come another day’ [Health worker, Mozambique].

**Figure 2.**
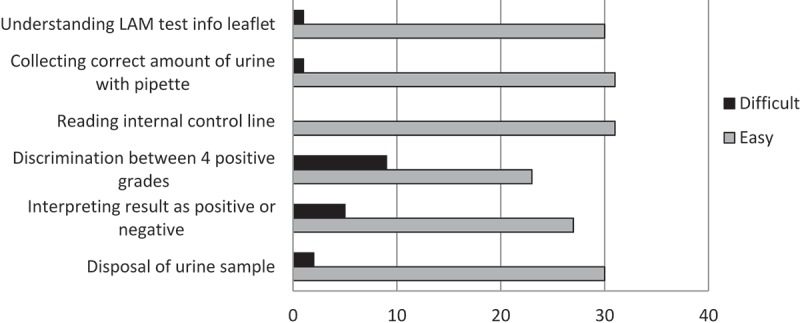
LAM users’ responses on the ease of performing each step of the LAM test (N = 32).

**Figure 3.**
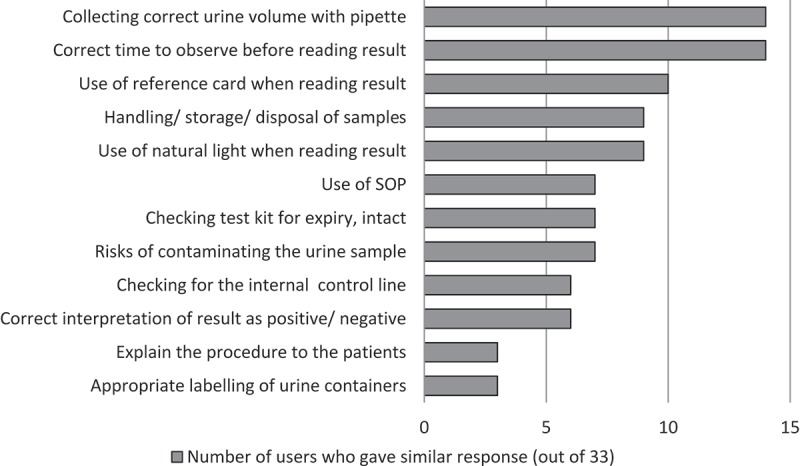
LAM users’ responses on the important elements to teach new users about performing the LAM (N = 32).

Many users found the LAM test easy to perform, requiring no special skill or qualification: ‘It is not complicated to train a person to carry out the LAM. You can even train HSAs (Heath Service Assistants) to do it’ [Laboratory technician, Malawi]. Responses to questions on the ease of performing the LAM testing procedure are presented in Figure 2. The most common responses concerning which steps of the LAM test procedure the users would reinforce during the training of new LAM users are given in Figure 3. Users performed a median of 3 (IQR 2–5) tests before they felt comfortable to perform it without assistance.

The LAM test was perceived as a welcome alternative to sputum-based TB diagnostic tests especially for patients who were not able to produce sputum: ‘It is useful in resource-limited places, were there is no laboratory for Gene Xpert and no chest X-ray available and is a great help for the asymptomatic patients’ [Health worker, Mozambique]; ‘The LAM test can diagnose TB even if the sputum result was negative and can also diagnose TB in patients that are not showing any symptoms’ [Doctor, DRC].

Four themes emerged from the analysis relating to the challenges of implementing the LAM test. First, challenges with LAM testing procedures; second, the lack of a clear protocol for LAM implementation; third, patient-related issues; and fourth, issues relating to the urine sample.

Several users had concerns regarding the reason for, as well as practicality of, using the reading card to discriminate between the four positive grades. Initially in DRC and in Mozambique, the reading card was not used and any line appearing in the patient window was interpreted as being LAM positive: ‘Using the reading card is not interesting because if it is positive it is positive. The result will not change back to negative’, [Doctor, DRC]. Some had difficulties in differentiating between Grade 1 and fainter-than-Grade 1 intensity.

Some users were concerned about the interpretation of a negative LAM result when there was a strong suspicion of TB. The fear of missing true TB patients was particularly palpable in DRC, where mortality was high: 30% of new admissions in the IPD, with overall 56% of deaths attributed to TB: ‘LAM is used but we should not rule out TB just because of a LAM negative result. If there is a high suspicion of TB the patient should get treatment. There is a real danger that LAM negative results will be used as a deterrent to anti-TB treatment’ [Program manager, DRC].

Other users placed little reliance on the LAM-positive results when CD4 was higher than the cut-off point for testing or when there were no symptoms of TB: ‘A female patient was seen in the IPD, presenting with fever, chest pain and night sweats. She was LAM positive and the CD4 result was 798 cells/µL. Part of the study team felt that it must be a false LAM-positive result since the CD4 count was high and the sputum microscopy and GeneXpert were eventually negative. The clinical officer however still maintained her on anti-TB treatment reasoning that the LAM result was a Grade 4 positive’ [Field notes, Malawi]; ‘If there are no clear symptoms and the LAM result is Grade 1, then I would not start TB treatment’ [Health worker, Mozambique].

It was observed in all three countries that users did not always wait for a CD4 result before performing the LAM test: ‘If the patient presents with clinical signs of tuberculosis or is in a generally bad state, then I do the test without waiting for the CD4 result’ [Doctor, DRC]; [Field notes Malawi]. The prior requirement of a CD4 result was considered as a challenge to LAM testing since the CD4 result TAT was reportedly long and was observed to vary from 2 h to 5 days: ‘If patients are asked to wait in another queue for CD4 testing they seem to get lost in the process’ [Field notes, Mozambique]

Despite the usefulness of the test in patients who could not produce sputum, a few users felt that there could be a reduction in patients’ compliance to submit other samples on time (e.g. sputum) if they had had a previous LAM test done: ‘There is lack of collaboration of some patients regarding the delivery time of the sputum after the LAM test’ [Health worker, Mozambique]. Some users also noticed that female patients could be reluctant to provide urine samples when menstruating, preferring to submit a sample only when the bleeding had reduced. Performing the LAM test in the consultation room was felt by some to lead to disruptions in the general flow of patients as patients brought in urine samples whilst other patients were being consulted. However, many did not perceive this as a major concern: ‘We tell the patients that they can just come in to leave the urine bottle. The other patients don’t mind. They know that next time it could be them’ [Nurse, Malawi].

Concerns emerged regarding contamination of urine during the collection by the patient and its handling by health staff: ‘No information was given to patients on how to collect the urine sample’ [Field notes, DRC]; There were also concerns related to the risk of misdiagnosis due to non-tuberculosis mycobacteria infections: ‘A question was raised by one staff during the study team meeting on how we really know if the LAM shows real TB or is reacting to another form of mycobacterium; this was during a discussion on the poor sanitary conditions in one of the clinic’s toilet facilities’ [Field notes, Malawi]. Some users were initially reluctant to believe the LAM test could truly identify TB, mistrusting urine as a suitable medium to perform a TB diagnostic test.

## Discussion

This is the first study to present findings on the feasibility of implementing the TB-LAM test under programmatic conditions in low resource settings. Our findings show that implementing the LAM test is feasible: it required minimal logistical input, it was easy to perform, and presented little extra workload for the users. Use of the LAM test made it possible for patients with a positive LAM result to start TB treatment immediately, although in some cases the slow turnaround time for accessing CD4 results negated this added benefit.

For patients who are LAM positive, using the LAM test reduces, in theory, the number of required visits to the clinic. Although we did not measure the actual number of visits, we believe that our deductions, also taking into account observations made by investigators whilst in the field, are representative of the true situation. A study in Tanzania found that patients in rural and urban settings made 3 and 2 visits, respectively, before a TB diagnosis was made by sputum-based methods [12]. According to health workers in Chiradzulu, the high cost of transport is the most frequently cited reason for failing to return to the health centre for scheduled appointments, and for failing to go to the district hospital for a chest x-ray. A South African study showed that the period leading to a TB diagnosis could lead to significant direct and indirect costs to the patients who often have limited resources to pay for these costs [13], further emphasizing the need for a test with same day results. Using the LAM could substantially reduce costs related to the pre-diagnosis period for patients who become LAM positive. Importantly, starting TB treatment on the same day of the test could also potentially reduce TB-related mortality [14,15].

Some of the challenges, including the initial confusion about the use of the reading card in DRC and Mozambique, the questions relating to usefulness of the reading card, and the challenges faced with interpretation of the LAM result, highlight the need to train staff appropriately before implementing the test particularly about the need to use the reading card and how to interpret the grading results. Challenges in the interpretation of a result as negative or positive may lead to misdiagnosis of patients, which may be harmful. The recommendation to the manufacturers of the Determine LAM test would be to consider simplifying the test by refining the titration points of the lines Grade 1 and fainter-than-Grade 1 or, better yet, to create a test with only one positive line. It is also essential to emphasize that patients with a negative LAM test can still have TB so the test should not be used as a rule-out tool as the sensitivity of the test is not optimal [16,17]. This notion should not be difficult to explain to staff familiar with sputum microscopy, since the interpretation of the results is similar.

The extra workload for staff from the LAM test can be considered small. In practice, the researchers observed that staff used approximately 15–20 min of the LAM test time to continue other activities, such as continuing with the patient consultation and examination. In the DRC context, each clinician had as many as three patients per day requiring a LAM test, translating to more time required in performing LAM testing for each clinician per day. In this and other similar high burden settings with limited human resources, LAM testing should be considered for less specialized health workers using strategies that maintain the short TAT for the test result. This is especially important if the health-care professionals feel that they have a high workload due to LAM, potentially leading to demotivation or staff burnout [18]. The excellent inter-reader agreement observed in our analysis implies that the LAM test is easy to use, even with the challenges to differentiate between the Grade 1 and fainter-than-Grade 1 reading. This further suggests that the LAM test could be performed by less specialized staff.

Our findings clearly show that the LAM result TAT is shorter when the test is performed at the point of care compared to when it is performed in a central place. Delays causing the longer TAT when LAM was performed in a central laboratory could be due to the time taken to transport urine samples to the laboratory especially in Malawi were the furthest clinic included in the study was 2 h away by car. In Mozambique, the laboratory was next to the consultation room and this may explain why there was no big difference in the TATs observed. Another delay to getting a LAM result from the laboratory could be due to the staff having other priorities, e.g. other ongoing tests. These results provide a strong argument to implement LAM during the individual patient consultation to achieve the maximum benefit. This ‘one stop’ approach may reduce the risk of patient loss-to-follow-up before the TB diagnosis is complete [19]. Delays in starting TB treatment after a positive LAM result could have been due to the fact that at all sites, initiation of TB drugs was done by a second staff, not the one who had prescribed the treatment. There were also delays due to administrative procedures before a patient was given the first dose (filling in of TB registers and collection of drugs from their storage). In DRC, the TAT and the time to first dose appears much higher compared to the other countries. An explanation could be that they attended to sicker patients who required more consultation time compared to those in Malawi and Mozambique.

The CD4 count is currently a criterion to select ambulatory patients eligible for LAM [20]. Given the concerns raised around the CD4 test turnaround time, it is crucial to design LAM implementation strategies that minimise the time to receive CD4 results, such as easy access to point of care CD4 machines. However, this will likely prove challenging or impossible as countries adopt the ‘treat all’ policy, which does not rely on CD4 testing as an indicator for antiretroviral treatment initiation. In Malawi, the MOH had stopped supplying CD4 testing material and CD4 testing was no longer available outside of study contexts. Introducing clinical criteria for LAM eligibility when the CD4 count is not available may be an intermediate solution, although this strategy risks missing some patients who could benefit from the LAM test [21]. The need for CD4 criteria in patient populations with a high risk of TB, such as hospitalized or ambulatory patients with symptoms of TB in high burden settings may also need to be reviewed.

The use of urine samples for this TB diagnostic tool is a particular advantage for severely ill HIV-positive patients who are unable to produce sputum [22]. Many more patients were able to provide urine than sputum, similarly to what has been previously described [23]. In DRC however, a substantial number of patients did not produce a urine sample compared to Malawi and Mozambique. The IPD where these patients were seen reported a high mortality (30%) during the period of the study. They received very ill patients and a lot of them died within a very short time (reportedly less than 24-h post admission) before a urine sample could be collected. In such a context, it would be relevant to further investigate the added value of using the urinary catheter to collect urine for LAM amongst very sick hospitalised patients. As there are other known limitations in detecting TB in sputum for HIV-positive patients [24], our findings emphasize the importance of non-sputum-based tests for HIV-positive patients to complement existing TB diagnostic options.

The findings of this study also emphasize the importance of proactive health messaging to avoid that the introduction of LAM increases non-compliance in submitting other samples, such as sputum, for TB diagnosis, as was the case in Mozambique. In addition, since the LAM test is sensitive to contamination from stool, soil and dust, which can lead to false positive results [25], it is important to clearly instruct patients on how to collect the urine specimen. Simple strategies on how to collect midstream urine specimens may go a long way in reducing the risk of urine sample contamination [26].

Although this study did not assess the costs of implementation, other studies have found it highly cost-effective to add LAM to TB diagnostic algorithms for HIV-positive patients [27–29].

This study has some limitations. First, our analysis was performed in health facilities that were supported by MSF, and thus may not accurately reflect outcomes in other public health facilities. In non-MSF supported facilities, staff workload may be higher due to the burden of more non-HIV-related activities. There may also be fewer or less available human resources (e.g. doctors and laboratory technicians). These constraints should be taken into consideration before encouraging a wider implementation of the LAM test. Second, there is a risk of response bias. Some health workers might have felt uncomfortable talking openly about some topics (for example, perceived workload), particularly if they had strong negative views about the program being studied or if they felt their employment might be affected by expressing those views. The research strategy sought to reduce this bias by implementing and communicating strict confidentiality measures, such as not using participant names or titles. Third, interviews were not audio-recorded because of resource constraints. We relied on interviewer memory and handwritten notes, thus risking the loss of some information. To minimize this risk, the interviewer completed the interview notes immediately after the interview was done. We also used triangulation, whereby more than one method of data collection was used to collect information on the same topic (e.g. we used written questionnaires in addition to the interviews). Finally, there was no assessment of patient perception of providing urine samples for the LAM. Instead, the study design relied on feedback from health workers on their interaction with the patients, which provided limited but important insight into how patients may have perceived the LAM and associated procedures. It is important to take into account patient perspectives relating to the submission of urine samples.

## Conclusion

The results of this study show that it is feasible to implement the LAM test in low resource settings. The test was successfully performed at the point of care: most patients were able to submit a urine sample, results were available in a very short time on the same day of the test, and the test required a minimal increase in clinician workload and no additional workspace to perform. The inclusion of the LAM test in the TB diagnostic algorithm could hasten the initiation of TB treatment for the most vulnerable patients. To maximize the test’s benefit, it is crucial to ensure adequate staff training on the use of LAM, including the use of the reading card, and on the interpretation of the results. In addition, the manufacturers of the LAM test could improve on the readability of the test results. It is also important to emphasize the need to continue submitting other samples for TB diagnosis, such as sputum. Finally, the requirement of a CD4 count as a criterion to identify patients who would benefit from LAM should be reviewed, while maintaining access to CD4 testing in the meantime.

## Paper context

In 2015, the Determine TB-LAM test was recommended as a tuberculosis diagnosis tool among HIV positive people. The accuracy of the test has been assessed in several studies, but there are still knowledge gaps related to its practical implementation. Our paper discusses the benefits and bottlenecks identified during programmatic implementation of the LAM test. These findings can guide policymakers and program managers as they consider wider use of the LAM test outside of study settings.

## Annex 1. Questions used in the one-on-one interviews

**Questions for those who have performed the LAM test**

- What is your profession, current work position?
- How long have you worked in this project?
- What do you know about the LAM test?
- For how long have you been performing the LAM test/when did you start using the LAM?
- Did you get any training to use the LAM?
  - Describe the training you received before you could perform the LAM test.
  - Who provided the training?
- Describe the process you follow when you consult a patient whom you think should have a LAM test done.
- Describe which patients are eligible for the LAM test (in the IPD/OPD).
- Is there a written protocol on how and when to utilize the LAM test? (Staff to show it or mention where it can be found).
- How many patients do you consult/look after each day?
- How many LAM tests do you perform per day?
- Where is the LAM test performed? (In the consultation room/bedside/laboratory?)
- Where are the LAM tests stored?
- What other equipment/supplies do you need to perform the LAM test (apart from the test strips themselves?)
- May you show how you perform the LAM test (in the consultation room/bedside)?
  - Where in the consultation room/bedside do you conduct the test?
- How long do you wait before reading the LAM test result (what you do in practise)?
- How do you dispose of the urine after performing the test?
- Do you give any information to the patients at the time you ask them to collect a urine sample?
  - Describe the information you give to the patients at the time you give them a container for urine submission.
- In your experience, do you consider the LAM test easy to perform, or difficult? Give your reasons.
- What are your views on performing the LAM test relating to your daily workload?
- Do you wait for a CD4 result before performing the LAM test?
  - Where are the CD4 tests performed?
  - Who performs the CD4 testing?
  - In your experience, how long does it take to get back CD4 results?
  - In your experience, what problems are in the CD4 testing process?
  - Are there delays experienced by patients in getting CD4 done? What causes these delays?
- Do you use the reading card?
  - Why don’t you use the reading card?
  - Did you ever start using the reading then stopped?
- In your experience, do the patients face any challenges in producing a urine sample?
  - What are these challenges?
  - What are the main reasons for patients not to produce urine, or to produce urine late?
- What are you views in relation to the introduction of the LAM test as a TB diagnostic tool?
  - Have you experienced any benefits of using the LAM? What are they?
  - Have you experienced any challenges of using the LAM? What are they?
- Do you have anything else to add with regards to the LAM test, or the TB diagnosis procedure?
- Describe the regular TB workout algorithm for patients seen in the IPD/OPD (the TB patient flow which does not include the LAM test). What happens when you suspect that a patient has TB?
- Describe the TB workout algorithm (which includes the LAM test) for patients seen in the IPD/OPD.
- Who collects the sputum samples?
  - In your experience, how long does it take before you get back sputum results (microscopy and GeneXpert?
  - In your experience, do the patients face any challenges in producing a sputum sample?
  - What are these challenges?
  - What are the main reasons for patients not to produce sputum?
  - Describe the process of how you send sputum samples to the district laboratory/the microscopist/another laboratory, and how you get back the result.
  - Are there any challenges you face during this process? What are they?
- In your experience, do the patients face any challenges in getting a chest x-ray done?
  - What are these challenges?
  - What are the main reasons for patients not getting a chest x-ray done?
  - In your experience, how long does it take before you get back chest x-ray results?

**Questions for other health staff involved in the TB diagnostic process**

- What is your profession/current work position?
- How long have you worked in this project?
- Describe the regular TB workout algorithm for patients seen in the IPD/OPD (the TB patient flow which does not include the LAM test).
- Describe what happens when patients are referred to you for sputum submission (for EHTs or microscopists).
  - How long does it take you to produce a microscopy result?
  - What causes the delays? Why are you not able to process the sputum the same day, for example?
  - Describe the process of how you send sputum samples to the district laboratory/another laboratory for GeneXpert, and how you get back the result.
  - Are there any challenges you face during this process? What are they?
  - In your experience, do the patients face any challenges in producing a sputum sample?
  - What are these challenges?
  - What are the main reasons for patients not to produce sputum?
- In your experience, do the patients face any challenges in getting a chest x-ray done?
  - What are these challenges?
  - What are the main reasons for patients not getting a chest x-ray done?
  - In your experience, how long does it take before you get back chest x-ray results?

**Questions for program managers**

- What is your profession/current work position?
- How long have you worked in this project?
- Describe the regular TB workout algorithm for patients seen in the IPD/OPD (the TB patient flow which does not include the LAM test).
- Describe the TB workout algorithm (which includes the LAM test) for patients seen in the IPD/OPD.
- Is there a written protocol on how and when to utilize the LAM test? (Staff to show it or mention where it can be found).
- Who performs the LAM tests? What is their profile? Why were they selected to perform the LAM test?
- When did you introduce the LAM test, and why was it introduced?
- What is the plan for rollout of the LAM test?
- What are you views in relation to the introduction of the LAM test as a TB diagnostic tool?
  - Have you experienced any benefits of using the LAM? What are they?
  - Have you experienced any challenges of using the LAM? What are they?
- What are your views on the LAM test relating to the daily workload of the health workers performing the LAM test?
- Do you have anything else to add with regards to the LAM test, or the TB diagnosis procedure?

## Annex 2. Ease of use written questionnaire- LAM test

**Date of response: … … … … / … … … … ./ … … … … .**

To be filled by each Laboratory technician, Nurse, Doctor, or CO who has performed the LAM test. Please complete the following sections on

**Please tick applicable**: Number of LAM tests performed**: <20 tests□ >20 tests□**

**Actual number of tests done if less than 20 (estimate): … … … … … .**

Please comment on how you found performing each of the following activities.
Question 1: Was the activity **difficult, OR easy**?
Question 2: Do you have any comment?

**Table:**

Item	Answer 1: Difficult = D; Easy = E;	Answer 2: Comments
Understanding the LAM test information leaflet		
Finding each of the items in the packet (test strips, reading card)		
Labelling the sample collection tube		
Setting the pipette to the correct volume		
Collecting the correct amount of urine using the pipette		
Pipetting the urine onto the sample pad		
Reading the internal control line		
Discriminating between the 4 positive grades by use of the reference card		
Interpreting the result as either positive or negative		
Disposal of the urine sample		
Disposal of the LAM test strip		
Disposal of the pipette tip		

***If you were training someone on performing the LAM test***:

What are the most important things you would teach them?

***If you were training someone on Reading the LAM Results***:

What are the most important things you would teach them?

**G – General comments**

At which steps of LAM testing procedure do you think there may be risks of errors?

After how many tests have you felt fully comfortable with the whole procedure (i.e. you were able to perform all steps without any hesitation)?

**•**In your own opinion, basing on your experience, what are the advantages of using the LAM test in the diagnosis of TB?

•In your own opinion, based on your experience, what were the main challenges in using the LAM test to diagnose TB?

**H – Any other comments?**

Date: ________________________________

Thank you for your feedback.
